# An Overview of Small Unmanned Aerial Vehicles for Air Quality Measurements: Present Applications and Future Prospectives

**DOI:** 10.3390/s16071072

**Published:** 2016-07-12

**Authors:** Tommaso Francesco Villa, Felipe Gonzalez, Branka Miljievic, Zoran D. Ristovski, Lidia Morawska

**Affiliations:** 1 International Laboratory for Air Quality and Health (ILAQH), Queensland University of Technology (QUT), 2 George St, Brisbane QLD 4000, Australia; tf.villa@hdr.qut.edu.au (T.F.V.); b.miljevic@qut.edu.au (B.M.); z.ristovski@qut.edu.au (Z.D.R.); 2 Australian Research Centre for Aerospace Automation (ARCAA), Queensland University of Technology (QUT), 2 George St, Brisbane QLD 4000, Australia; felipe.gonzalez@qut.edu.au

**Keywords:** air quality, UAVs, sensors, atmosphere, pollution, aerosols

## Abstract

Assessment of air quality has been traditionally conducted by ground based monitoring, and more recently by manned aircrafts and satellites. However, performing fast, comprehensive data collection near pollution sources is not always feasible due to the complexity of sites, moving sources or physical barriers. Small Unmanned Aerial Vehicles (UAVs) equipped with different sensors have been introduced for in-situ air quality monitoring, as they can offer new approaches and research opportunities in air pollution and emission monitoring, as well as for studying atmospheric trends, such as climate change, while ensuring urban and industrial air safety. The aims of this review were to: (1) compile information on the use of UAVs for air quality studies; and (2) assess their benefits and range of applications. An extensive literature review was conducted using three bibliographic databases (Scopus, Web of Knowledge, Google Scholar) and a total of 60 papers was found. This relatively small number of papers implies that the field is still in its early stages of development. We concluded that, while the potential of UAVs for air quality research has been established, several challenges still need to be addressed, including: the flight endurance, payload capacity, sensor dimensions/accuracy, and sensitivity. However, the challenges are not simply technological, in fact, policy and regulations, which differ between countries, represent the greatest challenge to facilitating the wider use of UAVs in atmospheric research.

## 1. Introduction

The composition of ambient air changes continuously, due to both natural and anthropogenic emissions which, when released into the atmosphere as aerosols or gaseous pollutants, affect air quality and human health [1]. The association between adverse health outcomes and poor air quality has been clearly demonstrated [2,3], and ambient air pollution has been recognized as the ninth largest health risk factor globally [4]. However, atmospheric pollution also reduces agriculture yields, visibility, sunlight at ground level and snowfall, and increases atmospheric heating as well [5,6]. These impacts highlight the need for continuous air quality assessment.

Detailed information on the characteristics of aerosol distribution and gaseous pollutant concentrations is needed when quantifying their effects on human health and the environment [7,8,9,10,11]. However, spatial and temporal resolution of data from ground, manned aircraft [7,12,13,14,15,16,17,18,19,20] and satellite measurements is relatively low and often inadequate for local and regional applications. In addition, satellite and airborne sensors can be prohibitively costly, restricting the use of these platforms to sporadic tests rather than routine analysis. Furthermore, taking measurements close to pollutant sources may not always be possible and it could be too dangerous or risky for manned aircraft to fly close to the ground [21,22,23,24]. Together, these reasons promote the use of small, lightweight UAVs for a range of applications, including atmospheric measurements.

Small, lightweight UAVs can provide more accurate information on aerosol distribution throughout the atmospheric column, which is needed to better understand air quality and composition in specific atmospheric layers [25,26,27]. UAVs cover large areas and can monitor remote, dangerous or difficult to access locations, increasing operational flexibility and resolution over land-based methods [28,29]. Since the application of UAV is relatively new, the aims of this review were to: (1) compile information on the use of UAVs for air quality studies; and (2) assess their major benefits and range of applications.

The review is organized as follows: the ‘Materials and Methods’ section explains how multiple bibliographic databases were used to search for published literature. The ‘Results and Discussion’ section describes the key aspects of UAV use which are critical for ambient air pollution monitoring [30], as well as an analysis of current UAV applications in air quality. The latter is divided into three keys areas of investigation: atmospheric composition, pollution and climate change; earth surface, interior and atmospheric phenomena; and prevention, patrolling and intervention. Finally, the current challenges, including possible solutions and future applications of UAVs, are presented.

## 2. Materials and Methods

This review used three different bibliographic databases, Web of Knowledge, Scopus and Google Scholar. Scopus provided extensive coverage of the topic area, but was limited to articles published after 1995. Google Scholar was useful for retrieving even the most obscure information, although its use was marred by inadequate or out-of-date citation information [31]. Different keywords were used and original peer-reviewed research articles and literature reviews were included in the search. In total, 61 search terms (See Table S1 in Supplementary Materials) and different combinations of these were used, including a combination of at least three individual terms. Data was compiled from published journal articles, conference proceedings, books and grey literature, namely technical reports published by government agencies, academic institutions, trade publications and information gathered from manufacturer websites, which are not typically subjected to peer review and may contain biased data. Although some cited reports came directly or indirectly from industries with a financial interest in promoting the use of UAVs, the data were cross-checked to ensure validity of the conclusions. The literature review ended in March 2016.

## 3. Results and Discussion

### 3.1. Air Pollutants Which Need to Be Monitored

#### 3.1.1. Atmospheric Composition

The composition of the atmosphere is strongly related to emission processes which release a wide variety of aerosols and gases, but are also connected to the Earth’s geography, meteorology and rotation which affect the movement of air masses [32,33]. The emission sources are both natural, such as vegetation, deserts, volcanoes and wild fires, and anthropogenic, such as power plants, domestic heating, transportation and industrial production. However, anthropogenic sources generally have a higher impact on health and the environment [34]. The main particulate matter (PM) and volatile organic compound (VOC) sources in urban environments are vehicle emissions, domestic heating and industrial processes.

Combustion processes release four principal greenhouse gasses (GHGs) into the atmosphere: carbon dioxide (CO_2_), methane (CH_4_), nitrous oxide (N_2_O) and halocarbons (gases with fluorine, chlorine and bromine) [35]. In addition, nitrogen monoxide (NO), sulphur dioxide (SO_2_), VOCs, PM, black carbon (BC) and organic carbon (OC) are also emitted [36,37].

#### 3.1.2. Health and Environmental Impact

Combustion-generated particles are the main contributors of PM in the urban atmosphere and they are strongly related to a number of adverse health effects [38]. Previous studies have identified that long-term exposure to combustion-related fine PM might be associated with an increased risk of cardiopulmonary and lung cancer mortality [39]. The PM emitted by anthropogenic combustion sources contributes a small fraction in terms of mass, but a very large number of particles, predominantly in the ultrafine range (Ultrafine particles (UFPs) < 100 nm) [40]. UFPs have a significant impact on human health and the environment [2,41]. They can penetrate and deposit deep inside the lungs [2,41,42] and because their lifetime is longer than particles with a larger diameter, they can be transported further from their sources, due to slow removal from the atmosphere [43]. In addition to their effects on human health, monitoring combustion-generated PM (including BC) is critical due to its capacity to adsorb sunlight, as well as its subsequent release of heat [44,45,46]. Assessing atmospheric VOC concentrations is also important, not only due to their direct effects on health, but also because they can act as precursors and become photo-chemically oxidized (atmospheric aging), leading to the formation of secondary organic aerosols (SOA), which have also been linked to poor health outcomes [47].

#### 3.1.3. Air Quality Measurements

Data collection in relation to gaseous pollutants, PM [48,49,50,51,52,53] and VOCs from traffic, power plants, urban and industrial air quality is extensive and includes ground level sampling using both on-line and off-line techniques [54,55,56], as well as manned aircrafts [57]. However, direct measurements at the source are not always feasible due to the complexity of sites, moving sources or physical barriers, such as direct measurements of shipping emissions or biomass burning.

It is critical to characterize in-situ air pollutant properties, in terms of origin, concentration mixing state, size, chemo-physical composition and reactivity, for both air quality and climate change research, as well as for policy development and the regulation of combustion source emissions. Therefore, UAVs may be a viable option for such in-situ air quality data collection [25].

### 3.2. UAV Types and Requirements for Outdoor Air Composition Studies

UAVs are operationally more versatile and visible compared to land-based approaches or other aerial methods, such as manned aircraft and satellites. Conducting atmospheric measurements in remote locations is one situation where the use of small, lightweight UAVs is of particular benefit [58,59]. In fact, the reduced size, weight and power needs of these flying robots, along with the reduced cost of the platforms and instrumentation, make them highly suitable for these operations.

**Figure 1:**
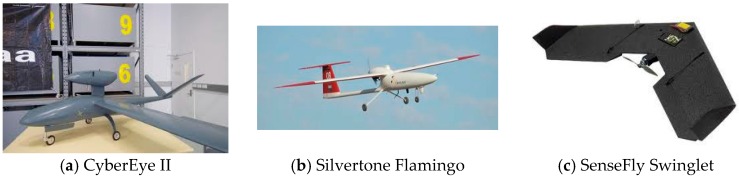

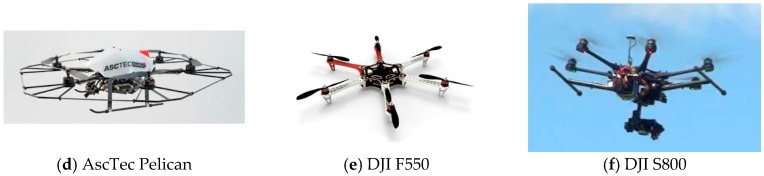
Example of a small fixed wing (**a**) CyberEye II [63]; (**b**) Silvertone Flamingo [64]; (**c**) SenseFly Swinglet [65] and rotary wing (**d**) AscTec Pelican [66]; (**e**) DJI F550 [67]; (**f**) DJI S800 [68] unmanned aircraft (All the UAVs shown are part of the fleet of the Australian Research Centre for Aerospace Automation).

Unmanned aircrafts encompass a wide range of different platforms which, due to their physical size and power, differ in terms of their capability and simplicity of operation. These factors impact the payload carrying capacity, speed, altitude and range of flight, which determines the different applications that can be performed by each type of UAV. Figure 1 shows examples of fixed and rotary wing UAVs. Several platform classifications have already been proposed, however, the nomenclature adopted for civil and scientific use has generally followed the existing military descriptions of size, flight endurance and capabilities [60,61,62].

Even though small UAVs are subject to significant payload restrictions compared to larger (manned) aircrafts, they have a distinct advantage over their manned counterparts in terms of relatively low platform cost, capability to perform autonomous flight operations from take-off to landing, and to fly closer to the ground with no risk for a manned crew [69]. Pre-programmed flight plans can be issued and automatically controlled on-board, which means that small UAVs can be flown with greater accuracy and less workload than aircraft with human operators. Some platforms even have the ability to work in environments without GPS signals and/or to follow local linear infrastructure without the use of a GPS [70], and therefore, could provide efficient and accurate monitoring inside buildings, forests or canyons [71].

#### 3.2.1. Performance and Capability of UAVs

UAV performance and capability are closely linked to aircraft size, and therefore, small, low-cost aircraft will inherently have payload, speed, power and endurance limitations. They will have a limited ability to carry on-board sensors/equipment and potentially short flight times. Airframe dimensions and shape, for example, can make the mounting of sensing equipment difficult, and power may have to be shared with the sensors, depending on the propulsion system. As such, large amounts of energy may be required, which will reduce both flight time and the spatial diversity of collected data. Low speed operation, governed by low stall speed characteristics, is often possible with such platforms, allowing for the spatially dense data collection often required for localized, site-specific inspections [72,73,74].

Larger aircraft have less stringent payload limitations and can accommodate an increased number and diversity of on-board sensors and equipment. A de-coupled propulsion and payload power system is typical, which further increases the potential for long-range, high endurance applications, such as large scale regional inspection. UAVs have been categorized into five groups by the U.S. Department of Defense (DoD), as shown in Table 1 [58].

To date, UAVs have been equipped with small size, lightweight visible-spectrum cameras or, in some cases, near-infrared cameras for conducting air quality measurements [60]. Chwaleba et al. [75] reviewed optical sensors that can be carried on-board a UAV for air pollution monitoring, ranking Light Detecting and Ranging (LIDAR) sensors as the best optical device to be used as a payload for air quality monitoring. Depending on the sensors used, multiple data sets may be collected with a high spatial and temporal resolution [60,76,77]. However, with more complexity and capability comes more maintenance, and additional specialist skills may be required. Larger platforms are costly and require a significant financial investment. Perhaps, the most important consideration is the safety of using such platforms in commercial applications, since they have the potential to cause considerable damage (to humans and property) and as such, fall under stricter operating guidelines than smaller UAVs [78,79].

#### 3.2.2. UAVs as Platform for Air Quality Research

Aircraft capability in the context of air quality monitoring is a critical aspect that needs to be considered, based on the purpose of the research. Fixed wing aircraft can typically cover a greater area over a given time interval and provide flexibility in terms of sensor mounting points. As they are unable to hover and have minimum operating height requirements, high spatial diversity can be achieved at the cost of decreased spatial resolution. In some cases, they can be inherently stable, allowing some forgiveness when failure occurs, such that payload recovery is likely. They may also require short runways (30–200 m), with some clearance for take-off and landing, or the use of a capture and release device [80,81,82,83]. They can be designed for compact and easy transport and deployment, allowing for operation in remote locations with minimal infrastructure requirements [84].

Rotary wing aircraft, such as helicopters and multirotor (quadrotors, hexacopters or octocopters), generally have a lower operating speed, but allow discontinuous trajectories, such as hovering, for close proximity inspection. Typically, increased spatial resolution can be achieved at the cost of decreased spatial diversity. Recent advances in control have made these inherently unstable platforms more reliable and easier to operate, reducing the risk of payload damage and accidents in general. They do not require specialized equipment or a runway for launch and recovery, and, depending on their size, can be easier to transport. Sensor placement has to be well matched to the platform, with the sensor intake typically located away from the propeller or rotor downwash effect.

Other aircraft types, such as parasails and blimps (balloons), can also be utilized [85]. They allow for larger payloads and slower operating speeds, compared to fixed wing aircraft. They may also have long operating times, but can, in some cases, be difficult to control and with less maneuverability [86]. This is mainly due to their high susceptibility to ambient weather conditions [87].

Unmanned aircraft systems can also be used in standalone operations involving a single platform, or more advanced systems utilizing multiple aircraft. In each case, a ground station is usually required for remote piloting and mission command. Multiple UAVs can be flown in a swarm, or coordinated to fly separately but with complementary trajectories for a given application [88]. This requires advanced centralized or decentralized control and guidance algorithms, but has the potential to increase the quality and quantity of data collected with a reduced operator workload. Currently, the use of multiple UAV systems has been demonstrated for a range of related applications [89].

Unmanned aircraft can also be viewed as useful tools for plume monitoring control and management within the disaster management framework [90,91]. They have the potential to provide high resolution spatial and temporal data sampling over large areas or in site-specific locations. They can also be used on a local, district or field level, depending on the type of data being collected and the platform characteristics. These data sets may then be coupled directly with other measurements, depending on the number of auxiliary sensors and aircraft/agents used. Cost per unit, including platform and auxiliary equipment, such as a ground station, may be significant (500–1,000,000 AUD), but in the appropriate circumstances, subsequent operation may be less expensive than manned aircraft operations or satellite based approaches.

#### 3.2.3. Regulations and Advisories

Importantly, unmanned aircraft cannot be deployed without restrictions. Under current aviation safety operating regulations, restrictions are placed on their use in commercial, research and private applications [92,93]. For example, in most countries, it is a requirement that UAVs be controlled by a certified operator. This adds an additional safety measure, but bears a cost and dictates who can legally conduct UAV operations [94]. This, in turn, has a direct effect on the frequency, quality and type of atmospheric research-related applications that can be conducted.

Considering that unmanned aircraft are flying robots operating in the same national airspace as manned aircraft, they too need to comply with governing aviation safety regulations. Aviation regulatory bodies, such as the Federal Aviation Authority of the U.S. (FAA) and Civil Aviation Safety Australia (CASA) define the rules and restrictions that govern who can access the national airspace and under what conditions, with the aim of protecting the general public and other airspace users by ensuring that safety standards are met. Some rules may only apply to a particular type of aircraft, while some may apply to all aircraft operating under certain weather conditions or flight types. Globally, a complete set of operating regulations for UAVs are yet to be defined [95,96].

### 3.3. UAVs for Ambient and Air Quality Composition Measurements

UAVs have been used by researchers and commercial organizations to sense atmospheric gases and aerosols [97], and have been shown to be capable of reaching remote areas and survey large regions [98,99]. The following sub-sections highlight the contribution of UAVs to the air quality research domain.

#### 3.3.1. Study of Atmospheric Composition, Pollution and Climate Change

The application of UAVs to measure atmospheric composition, pollution and climate change includes taking in-situ samples above, below and within the atmospheric boundary layer (ABL), as reported by a number of studies reviewed below.

##### Wind Vector Measurements

In order to measure wind vector, a UAV can perform a spiral flight trajectory. A Pitot tube mounted on the nose of a fixed-wing UAV could be used to measure and calculate the horizontal wind. However, no airflow angles are available, and so the wind vector can only be calculated by performing special maneuvers, giving a horizontal resolution of about 300 m [100].

A meteorological Mini-UAV $“M^{2}\mathrm{AV}”$ (5 kg takeoff weight, 1 kg payload, with more than 50 km flight range) capable of measuring T, H and wind vector data was developed by Spiess et al. [101,102]. The data collected by the UAV (temperature), were shown to be in good agreement, with a maximum difference of less than 0.5 K [100].

Van den Kroonenberg et al. [103] used the same UAV, the M^2^ AV, to develop a UAV system to collect wind data. The system had a five-hole probe (5HP), a GPS receiver and inertial measurement unit (IMU), meaning that an inertial sub-range of locally isotropic turbulence can be measured up to 40 Hz (or 0.55 m at 22 m·s^−1^ airspeed). During weak wind (3–4 m·s^−1^), the M^2^ AV data agreed with sonic detection and ranging (SODAR) and meteorological tower data to within 1 m·s^−1^.

However, UAV measurements were accompanied by large standard deviations of up to 0.4 m·s^−1^ when measuring stronger wind (6–7 m·s^−1^). The M^2^ AV measured higher mean wind speeds compared to nearby SODAR profiles, but agreed well with tower measurements.

The results of the work of Van den Kroonenberg et al. [103] suggest that to accurately measure the wind vector, any UAV would need a 5HP, GPS receiver and IMU.

Martin et al. [104] flew the $“M^{2}\mathrm{AV}”$ UAV during a campaign over Lindenberg, Germany, to measure morning and evening ABL transitions. With a climbing rate of 3 m/s and equipped with fast reading sensors (response frequency of 30 Hz), the $“M^{2}\mathrm{AV}”$ was able to provide a 10 cm vertical resolution for temperature, humidity, wind direction and speed. The collected data was in very good agreement (not larger than 1 K) with ground-based sensing systems.

A UAV called small unmanned meteorological observer (SUMO), was flown in Iceland during several campaigns to measure temperature, humidity, wind speed and direction up to 3500 m above ground level [88,105]. The SUMO operated successfully under polar conditions, reaching 1500 m in altitude, at a ground temperature of −20°C and a wind speed of 15 m/s. The results of these measurements were used to compare and evaluate the results of an Advanced Research Weather Forecasting (AR-WRF) model. The observed and modelled data were in good agreement; however, in some cases the model did not reproduce small inversions measured by the SUMO, which reported two layers at heights of 200–500 m and 1000–1300 m that were dryer than expected. In fact, the model overestimated humidity by around 25%. The collection of wind data was only possible during autonomous flight mode, which for safety reasons, could only be activated above 200 m, and therefore, resulted in no wind data being collected below that threshold. The need to operate under clear sky or thin cloud conditions was another limitation, since the SUMO stabilization system, which is based on infrared sensors, requires a radiation temperature difference of around 8 K between the ground and the sky [105]. Reuder et al. [88] worked on the optimization of the SUMO system to include: a new autopilot to enhance in-cloud flying capacity, a faster temperature sensor to reduce the measuring time from 5 to 1 s and by adopting the five-hole probe (5HP) system. The 5HP was the same approach suggested by Van den Kroonenberg et al. [103], which enabled the SUMO to determine 3D turbulent flow vector with a temporal resolution of 100 Hz.

The work of Reuder et al. [88] showed: (1) that was possible to integrate sensors onboard a UAV for air quality measurements (optimized within 650 flight missions since the first attempt in 2009 [106]); but also (2) that temperature, humidity and wind data, collected during a field campaign (Lannemezan, Franced) were significant for a case study on anabatic flow from the lowlands towards the foothills of the Pyrenees.

##### Atmospheric Aerosols Data Collection

The versatility of UAVs has been shown in several missions focused on the investigation of atmospheric aerosol properties, in particular light-adsorption and light-scattering properties. The application of a stacked configuration of three UAVs was successfully adopted during the Maldives Autonomous UAV Campaign (2006) to simultaneously measure aerosol-cloud-radiation parameters within the same column [99]. The aim was to clarify the nature of discrepancies between modelled and observed data under clear sky conditions. The UAVs flew with a horizontal separation of 10 m and a delay of less than 10 s to avoid ambiguities that arise from spatial and temporal variability in aerosols when passing over the same geographic point (or clouds). Results showed that the model was within an experimental error of 15% compared to the data collected when flying between 500 m and 3000 m. Both Ramana et al. [98] and Ramanathan et al. [99,107,108] stated that there was no need to invoke anomalous or excess absorption or unknown physics in clear skies.

The work of Ramanthan et al. [108] indicates useful information for sensor integration, flight path design and real flight of a UAV swarm. In addition to this, the work is significant in terms of data collected, overcoming the challenge of direct measurement of the solar heating caused by BC. This was possible thanks to the use of the three UAVs flown in a stacked formation over the same environment at different altitudes to measure flux divergences (heating rates) for an extensive period of time. The study also demonstrated that atmospheric brown clouds with a visible absorption optical depth as low as 0.02 are sufficient to enhance solar heating of the lower atmosphere by 50%. Corrigan et al. [25] used two twin fixed wing UAVs in a stack formation to monitor total particle mass concentration, particle size distributions, aerosol absorption and BC concentrations within the mixed layer over the Indian Ocean. Each platform had a weight of only 27 kg and 5 kg payload. A cruising speed of 60 knots (111 k/h) enables the UAVs to fly for up to 5 h, giving a nominal range of 550 km. The difference between data collected by airborne and ground instruments, and also aircraft-to-aircraft measurements, was <10%. However, measurements of aerosol optical depth taken by the UAV differed by up to 20% with those taken by a columnar AERONET sun-photometer. In this case, the data collected by the UAV allowed Corrigan et al. [25] to observe a large aerosol plume above the mixed layer, with a peak concentration near 2000 m. This result is in agreement with previous studies which disproved the common assumption that the mixed layer has a uniform concentration of constituents which decrease exponentially once above this layer.

The study by Bates et al. [109] aimed to generate a vertical profile of atmospheric BC concentrations, using a MANTA UAV in an 18-flight (38 flight hours) campaign. The payload was customized for online particle number concentration and aerosol light absorption (at three wavelengths) measurements, as well as particle collection using an 8-filter sampler (for off-line analysis). The flight plan for the UAV was to climb to 2700 m, descend to the altitude of maximum aerosol concentration, and then conduct sampling at that altitude. BC concentration varied from below the detection limit (0.04 μg·m^−3^) to 0.51 μg·m^−3^. Using the UAV, Bates et al. [109] were able to measure BC transport and distribution above the ABL, which was previously made with manned aircraft, being impossible to measure from a ground station.

Altstädter et al. [110] developed the ALADINA (Application of Light-weight Aircraft for Detecting In-situ Aerosol) system to investigate the 3D distribution of UFPs within the ABL. The UAV, equipped with two condensation particle counters (CPCs) and an optical particle counter (OPC), to give a total payload weight of 2.8 kg, was able to measure aerosols distribution from the ground up to 1000 m. The authors reported that the concentrations measured by the ALADINA were consistent with those obtained using a scanning mobility particle sizer (SMPS) system and an aerodynamic particle sizer (APS) at ground-level, however, the percentage of agreement between ground and airborne measurements was not presented. The work of Altstädter et al. [110] demonstrated the possibility of integrating two CPCs onboard a UAV to measure UPFs. However, the instruments measured the number of particles in the range of 11 nm and 2 μm and not only the UFP (<100 nm) fraction as stated in the title of the work. The research focused and provided details on integration of the sensors rather than validation of the data collected with the UAV system.

Harrison et al. [111] proposed the use of a remote-controlled aircraft to investigate the horizontal, vertical and temporal variability of PM within the first 150 m of the atmosphere. They aimed to prove that UAV-based systems could be the next generation validation methodology for satellites. Harrison et al. [111] used a modified 3 m fixed wing model aircraft (already successful in another study [112]) to perform four different flights: the first three followed an oval pattern with the altitude increasing after each loop, starting at 30 m and finally reaching up to 140 m. The UAV sensor package system was an aerosol spectrometer with the intake probe mounted in a cowl to allow clean, undisturbed air sample collection (payload specification not given). The mean PM_2.5_ concentration for three flights with varying altitude was 36.3 μg/m^3^, and the highest concentration was recorded below a 10 m altitude. Results showed an overall vertical variation with a standard deviation of only 3.6 μg/m^3^. The PM_2.5_ concentration did not change significantly across the day, with mean concentrations for the first three flights being 35.1, 37.2, and 36.8 μg/m^3^. A lower concentration of 23.5 μg/m^3^ was recorded during the constant altitude flight. Harrison et al. [111] believe that this reduction in concentration is significant compared to the concentration variations in any of the first three flights, therefore, that the constant altitude does not account for the change. For this reason, more data is needed to explain the cause of the change.

The flight at near constant altitude of 60 m was conducted to characterize variation on the satellite data subpixel scale; data was divided into a 120 m × 65 m grid. The mean PM_2.5_ concentration across the grid cells was within the standard deviation of any grid cell, with a variation from 20.5 to 24.9 μg/m^3^. This meant that the PM concentration could be accurately characterized as the data pixel area equivalent to the flight path area.

Other researchers have used multi-rotors to overcome the limitations of a ground-based station when measuring near-surface gradients of trace gases and PM concentrations. Brady et al. [113] used a quad-copter based system to study the vertical and horizontal concentration of CO_2_ and PM at a high spatial resolution (1 m) within the atmospheric mixed layer (0–100 m). A 3D Robotics Iris+ was used as a platform and the system made by integrating two commercial sensors onboard: (1) a dual channel optical sensor (MetOne 80080) to measure PM with an atmospheric diameter between 0.5 and 1 μm (channel one), and greater than 1 μm (channel two); (2) a NDIR CO_2_ sensor (CO_2_Meter-30) to detect CO_2_ between 0 and 10^3^ ppm. The payload weight was 510 g, limiting the flight time to 5 min, which was enough to observe sea-spray aerosol generation in the surf zone (a high-intensive production zone for sea spray aerosol due to the abundant wave breaking), showing high precision in both vertical (±0.5 m) and horizontal positions (±1 m). The UAV system was flown horizontally at 5, 10, 15 and 25 m from the beach to the surf zone to characterize the aerosol plume, providing both vertical and horizontal profiles. The profile, obtained from a total of 13 vertical samplings, demonstrated a maximum aerosol plume height of 40 m above the surf zone, and also a turnover in the 0.5–1 μm particle concentrations at an altitude of 70 m. These horizontal and vertical aerosol profiles enabled researchers to calculate ambient humidity emission rates for small and large particles in the surf zone. Future work aims to make the system lighter to increase flight endurance. Overall, the UAV system provided an efficient sampling platform to measure the vertical and horizontal profiles of sea spray aerosol generated within the ABL.

Mölders et al. [114] theoretically examined the capability of UAVs to provide spatial distribution of mean pollution concentrations using the 2009 Crazy Mountain complex fire in Alaska as a case study. The Evaluated Weather Research and Forecasting model in line with chemistry (WRF/Chem) data was used to represent ABL conditions. A virtual Scan Eagle capable of 20 h flight time (assuming zero-weight payload), was flown at different altitudes, speeds and following different path to collected data from the WRF/Chem. UAV measurements of CO correspond to the ground measurements within a factor of two at 1000 m, demonstrating that the UAV system can provide good 20 h mean distributions of CO at 1000 m for 60 km × 60 km. However, it is necessary to derive separate mean distributions for daytime and nighttime when considering pollutants involved in photochemical reaction chains (SO_2_, NO). In fact, results showed that the diurnal cycle of SO_2_ and NO concentrations led to overestimation compared to the ground measurements. Moreover, due to the relatively short period of darkness at high latitudes in late summer, a UAV swarm would be required for nighttime data collection. Each UAV would sample flying different pattern for 20 h.

Aviation advisory would benefit from the possibility of UAV to sample around the top of the ABL, which would give information on plume’s dispersion. This is particularly important when satellite imagery cannot provide such information due to cloud cover in the mid and upper troposphere.

Sampling strategies for meteorological and chemical quantities data collection are different. Air quality forecasts and the virtual sampling technique could help in effective, optimized flight planning, and data collecting. However, the authors assumed zero payload, while realistically even the lightest onboard sensors would add some weight. The heavier the payload the less fuel can be added, which reduces flight duration.

UAV deployment for air quality advisories requires long flight durations to cover large areas such as in the downwind of wildfires. Mölders et al. [114] asserted that instruments light and small enough to fit a UAV system capable of sampling at high frequency still have to be developed.

##### Greenhouse Gases and Other Gaseous Pollutants Measurements

Berman and Fladeland used a SIERRA UAV fitted with a custom-made GHG analyzer to conduct highly accurate (1 Hz) measurements of CO_2_, CH_4_, and water vapor concentrations at low altitudes (≥10 m) in Svalbard, Norway [115,116]. These SIERRA results are consistent with those measured by a Zeppelin station at 475 m above sea level (the percentage of agreement between readings at the different stations was not reported).

Malaver et al. [117,118,119,120] explored the possibility of flying a solar UAV as part of solar powered wireless sensor network system (WNS), to monitor GHGs continuously, using solar energy to solve the power consumption issue.

Rossi et al. [121,122] worked on the UAV and sensor energy consumption issue and successfully developed a new VOC and gas sensing system. The device is based on a fully automated metal-oxide (MOX) sensor, capable of running and storing data for 30 min. The researchers mounted the device underneath a hexacopter and performed two experiments. The first, hovering above an opened solvent (isopropyl alcohol) bottle, and the second, flying above the University refectory chimney, demonstrated that the sensing performance is not affected by the air and capability of the system to spatially describe VOC concentration.

Both the studies by Malaver et al. [117,118,119] and by Rossi et al. [121,122] were focused on sensors’ integration and illustrated the possibility of integrating low cost, low power consumption sensors to allow longer sampling times, for air quality applications rather than quantification of the measured gases.

Watai et al. [97] demonstrated that UAVs are a suitable and cost-effective method for measuring spatial and temporal variations of atmospheric CO_2_ in and above the ABL. The researchers integrated, calibrated and tested a 3.5 kg CO_2_ sensitive payload, with a 20 s response time and a precision of ±0.26 ppm. With a maximum flight time of 1.5 h, the UAV was able to rise to 2000 m (main observation area) and then descend in a spiral to about 650 m, before recovering and landing at the starting point. After 15 flight tests, Watai et al. [97] stated that the system was capable of conducting precise, high-frequency measurements, in order to determine the temporal trend of CO_2_ which tended to change between 200 and 400 m and 400–600 m layers. However, CO_2_ data were not collected at ground stations, and therefore direct comparisons were not possible.

Illingwoth et al. [123] confirmed both the cost efficiency of UAVs and the limitations of ground-based instruments to monitor the variability of target gases over a localized area as well as to provide important information in relation to the rapid characterization of micrometeorology and chemical composition. Illingwoth et al. [123] mounted an ozonesonde (manufactured by Science Pump Corporation., Camden, NJ, USA) on-board an inexpensive UAV (Skywalker Technology, Wuhan, China) to measure variations in ozone concentration on an urban scale. Flying close by Manchester, UK, they captured a peak concentration of approximately 39 ppm, which was associated with a short-term shift in wind direction. On the other hand, data collected by two nearby ground stations did not show this variance, but rather a constant O_3_ concentration of about 19 ppm and 26 ppm, respectively.

Lawrence et al. [124] developed a low-cost ( 400 USD airframe, 300 USD sensors) UAV system to address the emerging need for fine-scale measurements of atmospheric variables throughout the troposphere and lower stratosphere. The UAV system, DataHawk (0.7 kg, 1.0 m wingspan), was capable of in situ temperature, humidity, wind vector and turbulence sensing at high spatial resolution (1 m over a horizontal scale of 1 km), over altitudes ranging from a few meters up to at least 9 km. The work showed not only the possibility to integrate low-cost sensor to have accurate data (temperature, humidity and wind speed accuracy: 2 °C, 2%, 0.2 m·s^−1^), but also the ability of collecting data up to the top of the ABL.

Overall, UAVs can make significant contributions in terms of atmospheric data collection within the ABL, as well as in more complex environments such as mountainous areas [105]. Knowledge in relation to aerosol vertical profiles within the atmospheric column can also be improved when using UAVs, particularly where limited infrastructure is available, such as in remote or hostile areas. Furthermore, obtaining lower atmosphere profiles, up to about 3 km above ground, every 30 min, provides sufficient temporal resolution to investigate the relevant chemo-physical processes in the atmosphere. UAV systems could validate satellite data, and measure horizontal, vertical and temporal variability of gaseous and aerosol pollutants in the lower atmosphere, however, the intended application(s) of the systems have to inform its design. These design factors include the type of monitoring required (local, regional, etc.), the targeted activity required (VOC sampling, gases or data collection), operational frequency, safety, cost and long-term flexibility for that application and therefore the capabilities and limitations of the UAV platform.

Despite the cost and flexibility advantages, small UAVs are still limited by their short flight endurance, low payload capacity and network integration. Sensor technology is another limitation for the use of lightweight UAVs when fast sampling (1 s) and high resolution (ppb) are required. It is critical for researchers to select or design a new UAV system for air quality by taking into account how to validate system measurements, given that the sensors used in them may not have been originally developed to be mounted onboard a UAV. The use of small UAVs under a mass of 7 kg is favorable to maximizing their productivity. Using such class of UAVs, single flight duration and daily sampling are enhanced. The issue is that most commercially available, reasonably priced fixed wing systems are not designed to facilitate these operations. Set-up and breakdown times are excessive and individual components are not designed for heavy use [100].

These limitations need to be overcome, along with current aviation flight restrictions, in order to facilitate the extensive use of UAVs to assess air quality.

#### 3.3.2. Measurement of Surface, Interior and Atmospheric Phenomena

##### Volcano Emissions Data Collection

The limitations of using manned aircraft to conduct in-situ observation of volcanic plumes, which are transient and difficult-to-access, can now be overcome using UAVs.

In 2002, the first fixed wing UAV, an Aerosonde, carrying a miniaturized ${\mathrm{SO}}_{2}$ sensor [125] was freely flown for volcanic gas sampling [59]. Although the technical report did not show the specifications of the sensor or precise details of the analysis, researchers reported issues regarding the cost deployment, together with regulatory difficulties. At about that time (2000–2001), the Yamaha Corporation flew its latest unmanned helicopter to conduct surveillance imaging at the Unzen Volcano and at Mt. Usu, Japan [126], promoting the use of UAVs for the surveillance and investigation of natural disasters, such as earthquakes and volcanic eruptions.

Several years later, Astuti et al. [127] developed a novel “VOLCAN UAV system” for the measurement of volcanic plume chemical composition. This included the design of a new custom-made platform, an electric engine (required by volcanologists, in order to avoid instrument reading contamination, in the case of a gas engine), about 30 min of flying autonomy with a 3 km operational range (the distance between the plume and the base camp was roughly 2000 m), 5 kg payload capacity, a 40 km/h minimum cruise speed (to collect enough in-situ samples) and a maximum cruise altitude of 4000 m. A flight simulator (X-Plane), with a suitable plane model and interfaced with the real electronic boards, was adopted to simulate the UAV dynamics, in order to allow for easy tuning of control parameters [128,129]. However, based on time and money constraints, the researches decided to use a low cost petrol-powered model airplane (80 cm^3^, 2-stroke engine) instead, because the VOLCAN UAV still needed a proper launch system and real world tests before it could be used for the required measurements. The work of Astuti et al. [127] showed the possibility of integrating a gas sensor system onboard a UAV. However, the experiment focused on integration rather than quantification of trace gas species.

Longo et al. [130] went a step further and used data collected by robotic vehicles in the air and from the ground to design a new mixed Terrestrial Aerial Robotic PLAtform for Volcanic and Industrial Surveillance (TARPLAVIS). The TARPLAVIS system was comprised of several modules: a base station with a tele-control system, a six-wheeled Unmanned Ground Vehicle (UGV; ROBOVOLC model) equipped with different cameras for orientation and scenario screening (IR camera to thermally map the environment), a climbing robot ALICIA, and the VOLCAN UAV. Although the authors concluded that the potential and capability of this new multi-platform approach was tested for volcanic and industry surveillance, there are currently no data available in this regard. The aim of the study of Longo et al. [130] was to integrate different technologies in order to build a platform to collect data for industrial or volcanic emergencies. It was a success from an engineering point of view; however, it did not address the challenges of the application of the technology to real world capability as well as of the accurate sensor selection and validation.

In 2004, Saggiani et al. [131] flew a small fixed wing UAV (3.5 m of wingspan, 4 m long, max take-off weight 30 kg, 2 kg payload) around the Stromboli volcano (Italy). Three main issues were highlighted: difficulty finding a suitable area close to the volcano to use as a runway to take-off and landing (min 100 m); the inability to conduct a fully autonomous flight due to the limited UAV operational range (2 km max); and the limited payload capacity, which highlighted the need for a larger platform with better flight stability during bad weather. Based on the above considerations, Saggiani et al. [132] went on to test an INGV Raven UAV, which had a 6 kg payload (including an IR camera, a micro-interferometer (for O_3_ and H_2_O analysis) and a SO_2_ sensor), a flight range of 50 km and a flight time of 4.5 h. Although the researchers declared the mission a success, the data were fragmented, the landing system needed improving, and a ground control station was required to allow mission control from remote sites [133]. The use of UAVs for volcanology studies continued with the work of Amici et al. [134], who used a rotary wing UAV carrying an IR camera to thermally map a mud volcano (lower south-east flank of Mt Etna, Italy). Amici et al. [134] stated that the data from IR cameras at the ground level and those from the UAV were in good agreement, with the temperature of a cold pool detected during a flight found to be 34 °C by the UAV and 34.7 °C by the ground cameras.

Patterson et al. [135] flew an autonomous Silver Fox (lightweight UAV with a 9 kg take-off weight, max speed 110 km/h, service ceiling up to 4850 m) at an active volcano crater (Mount St Helens, Washington USA), delivering real time data to a remote location using visual and IR video sensors. The Silver Fox was also equipped with electro-chemical gas sensors (weight of less than 1 kg) capable of detecting seven different gases (authors did not provide further details). While this showed the potential for UAVs to provide useful information, even when using a relatively low-cost aerial platform, it was determined that both the sensitivity (1–100 ppm) and response time (30–60 s) of the sensors were insufficient in terms of range and reading speed to detect volcanic plume gases. The researchers highlighted the need for miniaturized sensors, as well as an optimized engine and airframe, to allow flights within acid and ash-bearing volcanic plumes, as well as close collaboration with scientists to improve the accuracy of data collection, interpretation and hazard analysis.

The potential of rotary wing UAVs for use in volcanology studies was confirmed by McGonigle et al. [136], who used an off-the-shelf platform (for less than 2000 USD) equipped with two different payloads below 2 kg: a UV spectrometer for SO_2_ monitoring and a multi-gas system for in plume analysis. The rotary-wing UAVs were flown less than 200 m downwind of the crater rim at Vulcano, Italy, following an eight traverses path underneath the plume to determine the SO_2_ flux. A reading error of 25% was estimated (±15% uncertainty from the spectral ${\mathrm{SO}}_{2}$ concentration retrievals, ±20% in plume transport speed measurement). After the first test, McGonigle et al. [136] used the second payload to measure gas concentrations by hovering the UAV inside the plume for 22 min, 10–100 m downwind of the crater rim. CO_2_ and SO_2_ were measured and the resultant CO_2_/SO_2_ ratio of 30 ± 5 was found to be in good agreement with the ratio of 35 found by Aiuppa et al. [137], who previously tested the performance of a portable gas analyzer at the same crater.

The study of McGonigle et al. [136] showed an innovative approach in addition to remote sensing, measuring CO_2_ and SO_2_ with a UAV from a volcanic plumes. The focus was not only on the sensor integration, but on the data analysis which showed the possibility of measuring gas concentrations in the plume downwind of the volcano. Collecting such data is normally very difficult due to SO_2_ cross-sensitivities of many gases and in particular O_3_. The study highlighted and detailed the possibility of using a combination of multiple sensors onboard a UAV, as an independent method for in-situ gas data collection.

It is critical to calibrate sensing systems and validate the data they produce, before mounting them onboard the UAV. Normally manned aircraft are used for this purpose, however, Pieri et al. [138] stated this approach in volcanology studies is an undue risk for the crew, even in dilute plumes (~1000–2000 μg/m^3^). The authors claimed that UAV observations proximal to the eruption site can provide the direct data collection necessary for model inputs and are more reliable compared to those currently collected from ground-based and satellite stations. The research suggested the use of three different UAVs: the Dragon Eye μUAV, the Wing Vector 100 and the SIERRA, and also highlighted the need for civil aviation authorities to recognize the unique benefits of UAVs for conducting measurements over volcanoes or within eruption plumes that are too dangerous for manned aerial vehicles.

Diaz et al. [139] worked to provide geochemical data using mass spectrometry techniques to investigate volcanic activity. The ULISSES is a small (10 kg weight and $21,000\;{\mathrm{cm}}^{3}$ volume), low powered (24 V) mass spectrometer (MS), developed in 2008 by Diaz et al. [140] in collaboration with NASA [141]. Continuing with their miniaturization studies for spectrometers, Diaz et al. [142] recently developed a MS with the world’s smallest turbo-molecular pump, to be mounted on-board a UAV for in-situ volcanic plume analysis. Two platforms were considered, the NASA Sierra UAV and the Wing 500 (3.3 m wingspan, 30 min of endurance and 4.5 kg payload), to carry the MS and other sensors for temperature, humidity, pressure, SO_2_, H_2_S and CO_2_. The MS was laboratory calibrated using H_2_, He, N_2_, O_2_, and CO_2_ gases at different concentrations (0 ppmv, 100 ppmv, 1000 ppmv and 10,000 ppmv). The UAV-MS system was tested in three locations: (1) Miravelles volcano, Costa Rica, where it found a CO_2_ concentration close to 75%, while H_2_S was 168 ppmv and SO_2_ was 24 ppmv; (2) La Bocca Grande fumarole, Italy, where the CO_2_ concentration was about 86%, the H_2_S concentration was 66 ppmv and the SO_2_ concentration was 13 ppmv; and (3) La Bocca Nuova fumarole, Italy, recording a CO_2_ concentration of 76%, and 68 ppmv and 11 ppmv for H_2_S and SO_2_, respectively.

##### Typhoons Data Collection

Atmospheric phenomena, such as typhoons, are another situation where UAVs have successfully been used to collect data to provide an independent check for ground-based measurements. In a study by Lin et al. [143], the Aerosonde was flown up to a height of 3000 m for a 10 h-long reconnaissance inside the eye of Typhoon Longwang. The measured wind speed was about 35 m/s, which increased to over 50 m/s for a period of more than 20 min and then dropped to less than 10 m/s, indicating that the Aerosonde was located inside the eye. The temperature measurements showed a more complicated structure when the Aerosonde flew inward, penetrating through the rain band and the eyewall along flight leg 1. At the convective region outside the eyewall (80–160 km radius), the temperature was higher at the 140 km radius, where the specific humidity was slightly lower. The temperature at a radius of 40–70 km (eyewall region) was slightly higher than that at the 80–160-km radius (the convective region outside the eyewall). The results pointed that the Aerosonde data, collected inside the typhoon, can serve as an independent check for Doppler radar wind retrieval.

##### Arctic Environment Data Collection

UAVs have also been shown to be effective for conducting environment and sea surface temperature studies in the Arctic [144,145]. Curry et al. [28] customized and tested the Aerosonde UAV in an industrial freezer (at temperatures down to less than −20 °C) to safely fly in the Arctic. The avionics were properly insulated, a fuel-injection engine was used to avoid carburetor icing and a servo-system was adopted to force ice breaking over the leading edge of the air-foil. Although icing and flight restrictions remain critical challenges, the Aerosonde demonstrated its capability in Barrow (Alaska), where three different Aerosondes provided continuous sampling for 48 h along a 30 ${\mathrm{km}}^{2}$ box. Curry et al. [28] reported that approximately 20 Aerosonde profiles flown at Point Barrow coincided with National Weather Service (NWS) soundings. The greatest variability occurred between 300 and 900 m, where the NWS sounding was 1–2 °C cooler than that obtained by the Aerosonde, probably due to a combination of different sonde instruments used by Aerosonde and NWS, and slight differences in locations of the soundings that could had given different results in a coastal environment. The aim of the study by Curry et al. [28] was to customize and integrate sensors onboard an Aerosonde to prove the UAV’s capability to fly in an hostile environment such as the Arctic. It was a success from an engineering point of view, however, the specifications of the payload used and a quantification of the error between the two measurement approaches were not provided.

Research into air pollution and atmospheric composition has already benefited from the use of UAVs, due to their flexibility and ability to access environments that are risky for manned platforms, such as volcanic plumes, typhoons and thunderstorms [146].

When sampling from a volcano plume, a rotary wing UAV would be particularly beneficial, as it can hover inside the plume [136], however, covering longer distances and higher altitudes to sense different atmospheric layers may be more suitable for a fixed wing UAV [133]. Physical, technological and regulatory limits as well as the best environments where UAVs can do the most, have no yet been identified. Defining environments where small UAVs can or cannot operate is still an open question (as with long coverage missions), but the integration into national airspace, i.e., regular, routine operations above 400 feet, emerges as the main issue. It is imperative for civil aviation authorities to acknowledge the potential of UAVs in order to facilitate their wide-spread use in atmospheric research and related scientific research in volcanology, typhoon study and meteorology.

#### 3.3.3. Measurements for Prevention, Patrolling and Intervention

UAVs can also be used for regular patrols around industrial areas [147] and metal ore sites. This for the purposes at air quality measurement extends the monitoring range beyond ground based fixed locations to collect/transmit data helping for faster decision making [148]. UAVs can also be used as a tool for the prevention and early diagnosis of environmental disasters, such as monitoring nuclear radiation levels in order to identify radiation leakage [149,150].

Alvarado et al. [151] developed both a small fixed wing and a multi-rotor UAV as part of a system to collect data after blasting at open-pit mines. The Teklite (commercially available) was selected for its portability, ease of integration of sensors, successful flight testing, light weight and low (<100 feet) target flight altitude. A custom made quad-copter of 2.5 kg (including batteries) was used to record data 35 m below ground level. The sensing payload, consisting of a SHARP GP2Y10 sensor to monitor PM_2.5_ and PM_10_, was calibrated in a lab, and demonstrated a high correlation (correlation coefficient R2 greater than 0.9) with an industry grade dust monitoring device. Talcum powder was used as the PM source to validate the system, with results showing the system to be capable of collecting significant data. Alvarado et al. [151] pointed out, however, that individual calibration equations are needed if the payload has different sensors, and that a different optical sensor is desirable to measure concentration with a precision higher than 1 mg/m^3^.

The research of Alvarado et al. [151] showed that integration of air quality sensor and an autopilot onboard a UAV is feasible. The UAV system could help to characterize airborne particulates in time and space, however, such system needs to be tested for real world application. Finally, an analysis of the data collected is required, to feed atmospheric modeling software and for flight path-planning algorithms.

Pollanen at al. [152] tested a new UAV payload below 0.5 kg, using a small gamma-ray spectrometer to detect alpha-particle emitting radionuclides in the air at the level of 0.3 Bq$/m^{3}$, assuming 0.5 h sampling and 1 h counting times. Pollanen at al. [152] used a low-active ${}^{137}Cs$ source (normally used for detector energy calibration) and autonomously flew the UAV over the unshielded sources several times within a time frame of approximately 50 s. The peaks were not well distinguished when flying above 100 m, since detection limits for the unshielded ${}^{137}Cs$ point source on the ground were approximately 1 GBq or larger, depending on the flying altitude. Significant data collection was possible at 75 m, demonstrating the capability of the Mini-UAV for aerial radiation surveillance, searching for lost or stolen unshielded point sources, and mapping radioactive fallout.

Hausamann et al. [91] proposed the use of UAVs to monitor oil and gas transmission pipelines, and investigated the use of two different UAVs for two different scenarios: the first using a small light-weight UAV for low altitude and high resolution data collection; and the second using a medium-size, medium-weight UAV, capable of carrying a heavier payload and with longer endurance. Although the results are yet to be published, Hausamann et al. [91] highlighted the need for a complete mission to determine the total operational capability of the system, including: the UAV platform, sensors, data processing and alarm detection. They also outlined the need for certification and operation standards, for the safe and efficient operation of UAVs. The work of Hausamann et al. [91] presented a comprehensive analysis of both the current technology useful for pipeline monitoring and the selection of two UAV platforms that could be used for such an application. However, questions of the real world capability as well as the integration and use of a UAV system for this application into national airspace still need to be addressed.

When fitted with pathogen traps and programmed for multi-location flights, UAVs can also be used for routine spatial and temporal data collection to facilitate early detection and prevent the spread of pathogens in the air [153], even in remote locations [154,155,156]. A multi-channel fluorimeter for real time fluorescent airborne particle detection was successfully integrated and operated on-board a UAV [157,158]. Similarly, Roldán et al. [159] developed a Mini-UAV to monitor atmospheric parameters while flying through greenhouses. Using a lightweight quadcopter (400 mm wheelbase, 125 mm square center), Roldán et al. [159] mapped CO_2_, T, H and luminosity profiles inside greenhouses. One of the challenges was to decide the most effective mounting point for the sensors on-board the UAV. For this reason, a computational flow dynamic (CFD) study was performed to confirm the findings of two previous quadrotor aerodynamic studies [160,161], which found maximum air speed at the rotor perimeters and minimum air speed at the center of the UAV and outside it, for both single rotor and quad rotor operation. Therefore, only two possible sensor mounting points were feasible: at the center of the UAV and some distance away from it. In order to confirm the results of the CFD study, Roldán et al. [159] attached the UAV to a cardan joint to maintain its precise location and, using an anemometer, they measured the air speed in a 24 point grid above and below the platforms, and found results consistent with the CFD simulations. A CO_2_ source was then placed on the ground and used to validate the system. The UAV flew at a height of 0.5 m, starting at 5 m away from the source. Results showed negligible relative errors for T (3.71%), H (1.65%) and CO_2_ (3.84%). Although the significance of the results was well explained throughout the paper, the same cannot be said for the sensor mounting point (in the UAV center), the height of the cardan joint, which CO_2_ source was used, and if any consideration was given to a possible ground effect. The results of a real-world test within a greenhouse (106 m × 47 m, 3 m height) showed an increase in temperature from the first measurement (25.3 °C) to the last one (29.6 °C), due to the transition from nighttime to daytime temperatures (from 9:00 a.m. to 9:22 a.m.) and the overall warming of the greenhouse. The humidity declined from 43% to 33%, while the ${\mathrm{CO}}_{2}$ concentration showed spatial variation, likely due to different ventilation efficiency in some areas of the greenhouse. Roldán et al. [159] stated that unlike UGVs, UAVs can collect data at nearly any point in the three dimensional space of a greenhouse, which facilitates activities such as local climate control and crop monitoring. This study showed the possibility of integrating cheap and light gas sensors onboard a small UAV. However, the study was focused on sensor integration and testing the system, rather than on quantification of the concentrations of the gases. Also, routine real world work might need longer flight times (the UAV used had less than 12 min endurance) and more than only 100 g payload capability.

##### UAVs as a Tool to Monitor Local Gas Emissions

Neumann et al. [90,162] examined the use of a gas-sensitive micro-UAV (200 g max payload, total take-off weight of 1.3 kg for 30 min flight time) to identify plume origins at ground level, as well as gas source localization and prevention. Neumann et al. [90,162] considered three different plume tracking algorithms: surge-cast, zig-zag and pseudo-gradient algorithms, and they performed a total of 5600 simulation experiments. The surge-cast algorithm was a combination of upwind surge and cross-wind casting. In this case, the UAV moved directly upwind into the plume and continued to travel for a distance, d, until it was no longer inside the plume. The UAV then flew cross-wind for a set distance (d cast), first on one side and then on the other, in an attempt to reacquire the plume. To maximize the chances of hitting the plume in the first cross-wind movement, the robot measured the wind direction to estimate from which side it left the plume. If the robot did not reacquire the plume by casting, the run was considered unsuccessful [163]. With the zig-zag algorithm, the UAV moved upwind at an angle, α, across the plume and when it sensed a concentration below a given threshold, the UAV was assumed to have reached the edge of the plume. It then re-measured the wind direction and continued moving upwind at an angle, α, with respect to the upwind direction. The UAV repeated this procedure moving in a zig-zag fashion within the plume, stopping when it reached the source. Finally, the pseudo-gradient algorithm included a new measuring strategy to deal with the strong disturbances induced by the rotors of a rotary-wing UAV, since measuring a local concentration gradient with spatially separated sensors as part of the gradient algorithm was not feasible in this case. The pseudo gradient algorithm also considered wind information in order to overcome the limitations of older gradient ascent methods that are unable to determine whether they are following a plume towards or away from the source. The simulation experiment results showed that the zigzag algorithm, with α=15° (where α is the angle used by the UAV to move upwind) and α=30°, as well as the surge-cast algorithm, were the most effective algorithms, but also the least robust. Higher robustness was shown by the pseudo-gradient-based algorithm and the zigzag algorithm with α= 60°. Subsequent experiments to determine the capability of the UAVs for real-world plume tracking were conducted using a ${\mathrm{CH}}_{4}$ bottle placed in the corner of a 20 × 16 $m^{2}$ area. A fan was used to blow the gas towards the UAV, which was placed 1.5 m away. The plume tracking experiments were stopped just after the UAV passed the source, but the algorithm was able to locate the gas source with a success rate of 83% ± 3%. Data collection behind the gas source would have also been advantageous to obtain a more accurate and reliable gas source location estimate. The study showed that it was difficult to locate gas sources in a dynamic environment where changes in wind speed and direction, together with high turbulence, were present.

Bennetts et al. [164] demonstrated the weakness of algorithms that directly mimic insect behavior, however, they proposed their use in source identification measurements where the plume is at a higher level, such as industrial chimneys, or where the airflow is more stable.

When using UAVs, and in particular rotary-wing platforms, the location of air sampler or air sensor intakes is crucial for accurate sampling [159].

Neumann et al. [165] described the results of experiments aiming to identify the best location for the gas sensor intake on-board a quad rotor UAV. Three different approaches were tested: active, using additional fans (12 V axial fan diameter 24 mm × 30 mm with an airflow of 5.4 $m^{3}/h$) connected to a tube going beyond the prop diameter; semi-active, using the airflow generated by the rotors; and passive, without any auxiliary device directing the airflow. Significant differences were found between the different gas sampling approaches, however, none of them were capable of measuring the reference gas concentration (0.5% by volume of CO_2_). The highest measured concentration was found using the active approach, which was 78% of the reference concentration. The average measured concentration was 0.33 ± 0.02 (active) % by volume, which was 66% of the reference concentration. Although the active gas transport approach was most effective at reducing the propeller dilution effect, the additional weight (76 g) of the long carbon fiber tube, the position of the tube inlet (which strongly dictates the measurement results) and the enlarged drone size (which makes it more susceptible to wind conditions) were found to be significant drawbacks. Gerhardt et al. [166] investigated the possibility of using a VOC sampler onboard a quadcopter, excluding the pump built into the device, and using instead the flow created by propellers to convey the air to the device. A steady flow rate above 150 mL/min was needed to remove the pump and the experimental results showed that sufficient flow rates are generated at the hover RPM (more than 80,000 times the minimum required for a reliable measurement).

Nathan et al. [112] investigated the possibility of using a 3 m fixed wing model aircraft as an airborne-based platform to quantify local emission sources to the atmosphere. The sensing payload consisted in a custom made laser-based open path methane sensor with a precision of 0.1 ppmv at 1 Hz, a mass of 3.1 kg and power consumption of 25 W. The UAV was an AMR Payload Master 100, modified to a dual-motor, each with 7.5 horsepower and 50 cm^3^ equivalent electric. The methane sensor was calibrated in a lab and compared to a LICOR LI-7700 before and after the flights. An uncertainty of airborne CH_4_ mixing ratio was estimated to be ±10%, normally insufficient for atmospheric measurements, but the nature of the mission means that the methane concentration above the background greatly exceeded this level of uncertainty. Data was collected from 22 flights around a natural compressor station to monitor CH_4_ leakage. The measured gas concentrations showed a high variability, from 0 to 73 (±40) g CH_4_ s^−1^, for hours to days. To understand if the reason was due to real changes in the emission rates, atmospheric variability or flight sampling issues, Nathan et al. [112] analyzed pairs of flights close in time and with similar weather conditions. The results highlighted an uncertainty due to flight sampling and atmospheric conditions based upon reproducibility at ±43% g·CH_4_ s^−1^. During the campaign, two additional and independent research groups measured the methane concentrations on the ground. Both of these independent measurements are consistent, but lower than the mean UAV measured rate of 14 (±8) g of CH_4_. This is likely due to the impossibility of measuring the lofted emissions by the ground stations.

Statistically, the interpolated methane downwind concentration was the main reason for the flux error analysis. In order to quantify this error, an interpolated map of areas where the flight did not sample is needed. The variance of the Kriged results at each grid point was assumed to be representative of the total uncertainty of the methane mixing ratio, and the standard deviation of the Kriged results gave a relative error of 46%. Nathan et al. [112] chose a 19 × 19 Kriging grid resolution to be consistent with the typical scales of individual plumes (10–30 m). The error due to the sensitivity of this grid resolution resulted in a 4% difference after the comparison with two other grids: 10 × 10 and 40 × 40, respectively. Gaussian-linear, Gaussian-cosine, linear and exponential were compared to the exponential-bessel variogram and no significant (5%) difference was observed. Based on this analysis, the overall statistical uncertainty of the emission rate was ±55% and for the median emission rate of 7.4 g CH_4_ s^−1^ is 4.1 g CH_4_ s^−1^, comparable to those estimated from the field derivate estimates (4.2 g CH_4_ s^-1^). The temporal distribution of fugitive CH_4_ emission rates from compressor stations needs further study, but the study highlighted that UAVs can be a valuable approach to quantify emission from point sources.

The UK Environment agency recently issued a report based on Allen et al. [167], who outlined the development of UAV systems equipped for GHG sampling with a focus on CH_4_ emission measurements from landfill sites. In this work a rotary (DJI F550) and a fixed wing (Bormatec Explorer aircraft) UAVs; operated successfully for 10 days. Allen et al. [167] ascertained that there are no high precision (<40 ppb at 1 Hz) CH_4_ instruments suitable for a small UAVs on the market. However, the results of the uncertainty analysis showed that measurement uncertainty is a very small component of the total uncertainty (at ~3% of the total flux). This would suggest that it may be possible to yield satisfactory flux uncertainties (<10%) with less precise CH_4_ instrumentation. An alternative could be to obtain CH_4_ measurements from a tethered rotary platform which involves vertical sampling (profiling) from a series of locations along a downwind transect. Suitable instruments for use with a rotary system and a sampling line are currently available (LGR-UGGA [168]). Using portable equipment (i.e., CH_4_ monitor in back-packs are commercially available) would overcome the long sampling time limitation required to move the system to different locations. Using a tethered UAS platform the size of the methane instrument would not be a constraint.

UAV systems have been demonstrated to be an appropriate platform for in-situ inspection systems to sample closer to the source. However, bias can be introduced in a number of ways, including sampling a region with significantly few pollutants and assuming a uniform distribution.

Quantifying emissions on a UAV is inherently challenging. A Gaussian single plume model is not ideal to create estimates of airborne data collection when there is more than one central plume. In fact, all basic Gaussian plumes would assume that constant and continuous emissions create a steady-state system. Therefore, the largest and most problematic assumption for these in-flight data sets would be to have one central plume. Although the point source approximation might be appropriate for most point source studies, the emissions from other locations around the investigated source could significantly be affected by the single-plume-model approach. To overcome this issue, researchers have investigated other approaches and the Kriging interpolation seems to be a better methodology to analyze UAV-acquired measurements from a point source [112]. Future studies need to optimize a more refined model that considers either a segmented Gaussian plume or a Gaussian puff. Currently, mechanical design limitations affect UAV applications. This is because the majority of commercially available UAVs are not designed to facilitate operations like quantification of CH_4_ emissions from compressor stations. These constraints can be addressed through UAV design, construction, and careful flight and sampling design [114]. Research should be directed towards effective mission/path planning, and validation targeted at ensuring unbiased sampling. Since most operations take place on controlled access sites, developing procedures that allow UAV use across the entire site during normal operational hours is required. Finally, payload and endurance limits, system network integration and aerospace legislation will affect UAV applications.

## 4. Current Challenges, Possible Solutions and Future Applications of UAVs

Air quality research is moving towards the more wide-spread use of UAVs to: measure gases at different altitudes; compare ground based data; and autonomously track plumes emitted by combustion sources, revealing the origin of pollutants [169]. New miniaturized instruments have been, and are still being developed, such as ultra-portable (10 kg weight and 21,000 cm^3^ volume) and low powered (24V) mass spectrometer (MS), for the study and visualization of gaseous volcanic emissions [140]. Similarly, research into UAV navigation is empowering more efficient bio-inspired algorithms [170] to enable aircraft to autonomously locate, and fly into or out of, an existing plume [171]. UAVs can complement existing wireless stationary networks (WSN) to improve the accuracy of data assimilation [90,172] by providing data comparable to that obtained using the stationary network, but with more than 30 sensors [173]. Table S2 in the Supplementary Materials summarizes the on-board UAV technologies which have been used for air quality data collection to date.

A recent literature review [174] suggested the use of UAVs to measure VOCs emitted from healthy plants and those under environmental stress. While this might enable researchers to detect plant stress and disease in its early stages, difficulties arise in terms of sampling methodologies and the chemical species to be sampled. Therefore, ground-based robots would be better suited for such an application, however, they could work in co-ordination with UAVs, to sample the broader area using optical sensors and relay information regarding site-specific points of interest. Alternatively, the sensor could be suspended from the UAV and a slow hover-like flight path would allow for the sensor to move through the field and obtain reliable measurements.

An analysis of the literature presented in this paper has highlighted potential applications for the use of small lightweight UAVs in the atmospheric research domain. However, there are a number of challenges that may prevent the science community from moving towards a widespread use of UAVs for air quality measurements [88,115,116]. The most significant of them relates to aviation policy and regulations that cover using unmanned aircraft to investigate atmospheric composition and phenomena in the ABL within national airspace systems [175]. In addition, limitations such as flight time, sensor integration and limited autonomy are driving research into the use of small UAVs. Table 2 shows the overall benefits and limitations and other challenges to the use of these small robotic platforms for air quality research.

Another potential research area is plume tracking using UAVs, but a system capable of such a task under real world conditions, including changing wind speed and direction, has not yet been developed. To date, experimental tests have shown that it is possible to track plumes where the airflow is more stable, like those emitted by industrial chimneys in a higher atmospheric layer, or on wide open landfill sites, geo-dynamically active regions or waste disposal sites [90].

Small instrument technology is developing quickly, with promising methods for both gas measurements, including tunable diode lasers, cascade quantum lasers and fiber chemical sensing [176], and PM measurements [177]. For example, a pulsed differential adsorption LIDAR (DIAL) system has been proposed as a potential UAV payload for atmospheric CH_4_ detection [178].

Future instruments for GHG analysis should also be equipped with on-board calibration cylinders for real time and in-flight instrument calibration [115], and autopilot improvements will enable more precise flight paths, as well as optimized sampling procedures [179].

There are no current high precision CH_4_ instruments (defined in the feasibility study as <40 parts per billion at 1 Hz) suitable for a small fixed wing UAS platform. Future methane instruments may also be suitable for rotary UAS platforms. The flight control and flight management software for multi-rotors is more mature than the equivalent software for fixed wing UAVs. The reason comes from both the requirement for sophisticated software to control multi-rotors and the current dominance of photography in the market for small multi-rotors UAVs. The result is that, for some applications, multi-rotors are the preferred solution; however, their lower endurance would preclude them from use on larger sites [100,167].

Established ground-based methods for air quality sampling, such as the sorbent tube or solid phase micro extraction (SPME) sampling techniques for VOCs, could be re-designed for use on-board UAVs, as well as integration with real-time display and data logging. Algorithm developments using multiple UAVs for plume tracking is possible where one UAV is flying upwind while the other tracks the plume downwind, in order to predict the path of the plume and provide early warning for areas of contamination. Genetics algorithms could also be used to obtain faster and more efficient reactions.

## 5. Conclusions

UAVs can offer high resolution spatiotemporal sampling, which is not possible or feasible with manned aircraft. Some UAV systems are more flexible than others, with the ability to carry multiple sensors and operate in different flight modes (hybrid rotary/fixed wing designs).

The future of UAVs for use in air quality applications is promising, thanks to the capability and flexibility of these robotic platforms. At the same time, new technologies in fields such as chemistry, physics and information technology are also developing very fast, resulting in smaller and lighter devices, with higher sensitivity and the ability to work remotely.

Questions still need to be addressed regarding the miniaturization of sensors, which seems to be the main issue when working with lightweight UAVs. In fact, the diverse range of UAV payload capacity is primarily made by the difference between rotary and fixed wing UAVs. The key limiting factors in new sensor development include: power, mass, and size, because these are intimately connected to the type of platform (rotary instead of fixed wings), engine (electrically or gas powered) and the type of mission (speed, longer distances with low altitude, rather than flight stability and low speed for a higher spatial resolution). Strict civil aviation regulations mean UAVs are commonly deployed for weather forecasting, atmospheric monitoring or as a tool for volcanology research.

## Figures and Tables

**Table 1:** UAV categorization used by the U.S. Department of Defense [58].

UAV Category	Max Takeoff Weight (Gross)	Normal Operation Altitude (ft)	Airspeed
Group 1	<20 pounds	<1200 (365.76 m) above ground level (AGL)	<100 knots
	(9.07 kg)		(<185.20 km/h)
Group 2	21–55 pounds	<3500 (1066.8 m) AGL	<250 knots
	(9.53–24.95 kg)		(<463.00 km/h)
Group 3	<1320 pounds	<18,000 (5486.4 m) mean sea level (MSL)	Any airspeed
	(<598.74 kg)		
Group 4	>1320 pounds		
Group 5		>18,000 MSL	

Note: if an UAV has one characteristic of the next higher level, it is classified as being part of that group.

**Table 2:** Overall benefits and limitations for the use of small lightweight UAVs in the atmospheric research domain.

Benefits	Limitations
Cost—small lightweight platforms are less expensive vs. manned aircraft, ground based instruments and satellites	Endurance limitations—flight time is still one the greatest limitations
Flexibility—wide range of UAV applications for atmospheric research	Payload capacity
Time—deploying a small UAV platform saves time vs. large manned platforms as well as ground stations	Sensor availability—limited choice for professional sensors suitable for mounting on-board a small lightweight UAV
Safety—there is no risk for crew when flying UAVs in dangerous situations such as close to the ground	Sensors limitations—smaller sensors may have less sensitivity, selectivity
Repeatability—ground station allows following the same programmed flight path every time	Aerospace regulation—a complete set of UAV operating regulations has not yet been globally defined
Routine flights—data collection for routine flights can be tedious/stressful for humans	Civil aviation authority recognition—UAVs has unique benefit for air quality research, over volcanoes or within eruption plumes
Dirty environments—UAVs can fly in dangerous environments such as when contaminated by radiological, biological, chemical hazards or volcanic plume	System network integration
Easy to deploy—small UAVs do not need airport runways, with fixed-wing UAVs able to take-off in less than 10–30 m while rotary-wing UAVs do not need runways	Autonomous plume tracking—although few algorithms for autonomous plume tracking have been developed and successfully tested in simulation environments, the real world feasibility still needs to be proved
Data collection—small UAVs can take measurements at any point in three dimensional space	
**Challenges**	
Overall deployment cost—to allow more dangerous missions where there is the risk of losing the UAV	
**Public Perception**	
High resolution 4D (time & space) atmospheric data sets—with the use of UAV swarms performing simultaneous operations. These goals will assist in providing adequate flight time for various integrated and time series data collection missions, real-time data links and imaging, and also to develop autonomous methods for sample collection	
Comprehensive in situ chemo-physical characterization of combustion source emissions, (including, UFPs, VOCs and above mentioned compounds)	

## Supplementary Materials

The following are available online at http://www.mdpi.com/1424-8220/16/7/1072/s1, Table S1: Search terms, Table S2: Summary of the lightweight UAV onboard technology for air quality research.

## Abbreviations

The following abbreviations are used in this manuscript:

UAV	Unmanned Aerial Vehicles
UGV	Unmanned Ground Vehicles
GHGs	Greenhouse Gasses
PM	Particulate Matter
VOC	Volatile Organic Compound
UFP	Ultrafine Particle
BC	Black Carbon
OC	Organic Carbon
FAA	Federal Aviation Authority of the U.S.
CASA	Civil Aviation Safety Australia
WSN	Wireless System Network
NASA	National Aeronautics and Space Administration
MOX	Metal Oxide
NDIR	Non-dispersive Infrared
SPME	Solide Phase Microextraction
