# Supplementary material for: An Overview of Small Unmanned Aerial Vehicles for Air Quality Measurements: Present Applications and Future Prospectives
Source: Sensors (Basel). 2016 Jul 12;16(7):1072. doi: 10.3390/s16071072 (PMC4969839; doi:10.3390/s16071072)
Supplement: Supplementary file 1 [file sensors-16-01072-s001.pdf]

Supplementary Materials: An Overview of Small Unmanned Aerial Vehicles for Air Quality Measurements: Present Applications and Future Prospective

Tommaso Francesco Villa, Felipe Gonzalez, Branka Miljievic, Zoran D. Ristovski and Lidia Morawska

Table S1. Search terms.

Aerosol	Airborne	Aircraft	Ambient	Environment
Air	Composition	Compounds	Satellite	Ground
Atmosphere	Approach	Methodology	Manned	Impact
Combustion	Source	Plume	Sensing	Sensor
Emission	Reference	Concentration	Climate	Sensing
Health	Outdoor	Method	Platform	Factor
Station	Data	Collection	Measure	Measurements
Trap	Onboard	In-situ	On-line	Atmospheric
UAV	UAS	Unmanned	System	Sampling
VOCs	Gases	Gasesous	Pollution	Pollutant
Drone	Remote	Control	Model	Hexacopter
Quadcopter	Multirotor	Tethersonde	Plane	Helicopter
UAR				

Table S2. Summary of the lightweight UAV onboard technology for air quality research.

UAV Name	Onboard Technology	UAV Weight (Kg)	UAV Payload Weight (kg)	Targeted Analytees (Gases, VOCs)	UAV Endurance (Minutes)	UAV Dimensions
[109] Kite Plane, Sky Remote	Non-Dispersive Infra-red (NDIR) gas analyser (LI-800, LI-COR)	16	3.5	atmospheric CO_2	Typically 60 but it can be extended until 90	224 cm × 308 cm × 119 cm (length × width × height)
[134] Thunder Tiger Raptor 90 Helicopter	Ultraviolet spectrometer<br>Infrared spectrometer<br>Electrochemical sensor	3.5	3	SO_2 flux<br>CO_2/SO_2 ratio<br>CO_2/SO_2 ratio	12	141 cm × 46.5 cm × 19 cm
[125] DP Volcan	Infrared spectrometer<br>Electrochemical sensor	13	5	CO_2SO_2	30	Wing span: 239 cm<br>Height: N/A<br>Length: N/A

Table S2. *Cont.*

UAV Name	Onboard Technology	UAV Weight (Kg)	UAV Payload Weight (kg)	Targeted Analytees (Gases, VOCs)	UAV Endurance (Minutes)	UAV Dimensions
[117,118] SIERRA	Laser gas analyser Off-Axis ICOS	180 (gross take-off weight)	19.5	H_2O, CO_2, CH_4	480–600	Wing span: 609.6 cm (20 feet)<br>Length: 309.66 cm (11.8 feet)<br>Height: 140.21 cm (4.6 feet)
[169,170] T-Rex Align 700E Helicopter	Vertical cavity surface emitting laser (VCSEL)-based sensors	4.7	5	CO_2, CH4, H_2O<br>CO_2, CH4, H_2O	20	Length:133 cm long, Height: 41 cm<br>Main rotor diameter: 156 cm
[115] MANTA	Mixing condensation particle counter (MCPC)<br>Three-wavelength absorption photometer (BMI ABS)<br>Multi-Channel Chemical Filter Sampler	16.3	11.4 maximum	Atmospheric aerosol not used	270	Wingspan: 270 cm<br>Height: 62 cm<br>Length: 192 cm
[119] Skywalker	Electrochemical Concentration Cell (ECC)	3 (max take-off weight)	N/A	Ozone	60 minimum	Wingspan: 188 cm<br>Height: N/A<br>Length: 110 cm
[33] Manta	Optical particle counter (OPC)<br>Aerosol absorption photometer	27 (max take-off weight)	5	Total particle concentration (number/cm^3)	270	Wingspan: 270 cm<br>Height: 62 cm<br>Length: 192 cm
[159] Patria MASS mini-UAV	Gamma-ray spectrometer (Georadis RT-10)	3	0.5	gamma-ray emissions	60	Wing span: 150 cm<br>Height: N/A<br>Length 105 cm

Table S2. *Cont.*

UAV Name	Onboard Technology	UAV Weight (Kg)	UAV Payload Weight (kg)	Targeted Analytees (Gases, VOCs)	UAV Endurance (Minutes)	UAV Dimensions
[120,122] Mini-UAV (M2AV)	5-hole probe for wind speed and direction measurement	6 (max take-off weight	1.5	Meteorological information	50–60	Wing span: 200 cm
	Vaisala hmp 50 for temperature and humidity measurements					Height: N/A
	Thermocouple for measuring fast					Length N/A
	Temperature fluctuation					
[36] Aerosonde	Sulphur and Carbon compounds detector	13–15	2	Cloud physics SO_2, CO_2	>than 30 h	Wing span: 290 cm<br>Height: N/A<br>Length N/A
[131] RAVEN UAV	Fourier micro-interferometer based on MOEMS technology	56 (max take-off weight)	N/A	Atmospheric trace gases	N/A	Wing span: 460 cm<br>Height: N/A<br>Length: 365 cm
[141] Aerosonde	Vaisala RSS901 PTU sensor (fast-response pressure-temperature-humidity sensor)	13–15	2	Pressure, Temperature and Humidity measurements	>than 30 h	Wing span: 290 cm<br>Height: N/A<br>Length N/A
[104,146] Airrobot AR100-B microdrone	Drager X-am 5600 gas detector, which include: catalytic, electrochemical and infrared sensors	1.3	0.2	Combustion gases and other toxic gases	20–30	Diameter: 100 cm
[136] THE DRAGON EYE μUAV	Small electrochemical SO_2 sensor<br>Small optical particle counter (0.5–5 μm range)<br>Small evacuated vacuum sampling bottle (0.1deci-liter) with an automatic actuator<br>Temperature-pressure-humidity sensors		<0.5 kg	SO_2, t, p, %H_2O, Gps, opc, nanopc, Color vis, lo light Vis, thermal ir, Evacuated Sampling bottle	N/A	Wing span: 114 cm<br>Height: N/A<br>Length N/A
[136] Vector Wing 100	Small electrochemical SO_2 sensor<br>Temperature-pressure-humidity sensors	3.6	<1 kg	SO_2, t, p, %H_2O, Gps, color vis	N/A	Wing span: 210 cm<br>Height: N/A<br>Length N/A

Table S2. *Cont.*

UAV Name	Onboard Technology	UAV Weight (Kg)	UAV Payload Weight (kg)	Targeted Analytees (Gases, VOCs)	UAV Endurance (Minutes)	UAV Dimensions
[136] SIERRA	Two laser altimeters<br>Synthetic aperture radar (sar)<br>Zenith- and nadir-pointing micro-spectrometers<br>Digital still and video tracking cameras<br>Pyrometer<br>Zenith pointing<br>Pyronometer<br>Also tested on SIERRA UAV:<br>TSI Inc., Shoreview, Minnesota, USA Model 3007 particle counter<br>TSI Inc. Model 3330 Optical Particle Spectrometer<br>Droplet Measurement Technologies<br>Mini-nephelometer—to obtain particulate scattering coefficients<br>Aerosol Drum Impactor<br>Bolometer<br>Temperature, pressure & relative humidity<br>Hal Technology (haltech), LLC HPC-600 six-channel hybrid handheld particle counter<br>Electrochemical SO_2 sensor<br>ULISSES mass Spectrometer	180 (gross take-off weight)	<45 kg	SO_2, t, p, %H_2O, Gps, opc, nanopc, Color vis, lo light Vis, thermal ir, Evacuated sampling bottle	480–600	Wing span: 609.6 cm (20 feet)<br>Length: 309.66 cm (11.8 feet)<br>Height: 140.21 cm (4.6 feet)
[133]Silver Fox	Visible electro-optical and IR cameras<br>Gas sensors	9.1 (20lb)	3.6 (8lb)	Thermal and visible images 7 different gases (no further information are provided by the authors)	10 to 20 h	Wing span: 244 cm (8 feet)<br>Height: N/A<br>Length N/A
[166] Aerosonde	Vaisala RSS901 radiosonde<br>Infrared gas analyzer<br>UV ozonesonde<br>Conductometric detector<br>Electrochemical detector	15	5 (2 of fuel)	Pressure, Temperature & humidity H_2O, CO_2O_3CO, CO_2, SO_2	>than 30 h	Wing span: 290 cm<br>Height: N/A<br>Length N/A

Table S2. *Cont.*

UAV Name	Onboard Technology	UAV Weight (Kg)	UAV Payload Weight (kg)	Targeted Analytees (Gases, VOCs)	UAV Endurance (Minutes)	UAV Dimensions
[171] Green Falcon	Metal oxide (MOX) nanowires gas sensor	5	<500 gr	NH_3NO_2	30	Wing span: 3.6 m<br>Length 1.3 m
[151] Senior Telemaster (ST) model airplane	Petri dish lid 2 × 100-mm	N/A	N/A	Air samples	N/A	Wingspan: 240 cm<br>Height: N/A<br>Length N/A
[153] Flamingo	Spore trap	12–15	10	Air samples	420	Wingspan: 400 cm<br>Height: N/A<br>Length: 290 cm
[116] CAROLO P360	Optical particle counter OPC (model GT-526, Met One Instruments Inc., Washington, DC, USA) O.M. two condensation particle counters, CPC (model 3007, TSI Inc., Shoreview, Minnesota, USA)	25 (max take-off weight)	<2.8 kg	UFPs number concentration of particles with different diameters<br>OPC: diameters between 0.3 to 10 μm<br>CPC: diameters between 11 nm and 2 μm (N11)<br>PC: between 18 nm and 2 μm (N18 25)	40	Wingspan: 3.6 m<br>Height: N/A<br>Length: N/A
[156] Parrot AR. Drone 2.0	Temperature/Humidity sensor (RHT03)<br>CO_2 sensor (MG811)<br>Luminosity sensor (TSL2561)	0.420 (take-off)	N/A	CO_2 concentration<br>Air temperature and humidity<br>Solar radiation	N/A	Wheelbase: 51.7 cm
[100] AMR Payload Master 100	Custom laser based open path methane sensor	N/A	N/A	CH_4, temperature, pressure, wind speed and direction	N/A	Wingspan: 335 cm<br>Length: 244 cm
[99] AMR Payload Master 100	Grimm 1109 aerosol spectrometer	N/A	N/A	PM_1, $PM_{2.5}$, PM_{10}, temperature, pressure, humidity, airspeed	N/A	Wingspan: 335 cm<br>Length: 244 cm
[101] 3D Robotics Iris+	Optical particulate counter MetOne 80080 two-channel particle counter.<br>CO_2 Meter K-30	1.282	400 g	PM 0.5 μm < d_P < 1 μm (channel 1),<br>PM d_P > 1 μm (channel 2), CO_2	16–22	Wheelbase 55.5 cm
